# Th1-Th17 Cells Mediate Protective Adaptive Immunity against *Staphylococcus aureus* and *Candida albicans* Infection in Mice

**DOI:** 10.1371/journal.ppat.1000703

**Published:** 2009-12-24

**Authors:** Lin Lin, Ashraf S. Ibrahim, Xin Xu, Joshua M. Farber, Valentina Avanesian, Beverlie Baquir, Yue Fu, Samuel W. French, John E. Edwards, Brad Spellberg

**Affiliations:** 1 The Division of Infectious Diseases, Los Angeles Biomedical Research Institute at Harbor-University of California at Los Angeles (UCLA) Medical Center, Torrance, California, United States of America; 2 The David Geffen School of Medicine at UCLA, Los Angeles, California, United States of America; 3 Laboratory of Molecular Immunology, National Institute of Allergy and Infectious Diseases, National Institutes of Health, Bethesda, Maryland, United States of America; 4 The Department of Pathology, Harbor-UCLA Medical Center, Torrance, California, United States of America; 5 The Division of General Internal Medicine, Harbor-UCLA Medical Center, Torrance, California, United States of America; University of Birmingham, United Kingdom

## Abstract

We sought to define protective mechanisms of immunity to *Staphylococcus aureus* and *Candida albicans* bloodstream infections in mice immunized with the recombinant N-terminus of Als3p (rAls3p-N) vaccine plus aluminum hydroxide (Al(OH_3_) adjuvant, or adjuvant controls. Deficiency of IFN-γ but not IL-17A enhanced susceptibility of control mice to both infections. However, vaccine-induced protective immunity against both infections required CD4+ T-cell-derived IFN-γ and IL-17A, and functional phagocytic effectors. Vaccination primed Th1, Th17, and Th1/17 lymphocytes, which produced pro-inflammatory cytokines that enhanced phagocytic killing of both organisms. Vaccinated, infected mice had increased IFN-γ, IL-17, and KC, increased neutrophil influx, and decreased organism burden in tissues. In summary, rAls3p-N vaccination induced a Th1/Th17 response, resulting in recruitment and activation of phagocytes at sites of infection, and more effective clearance of *S. aureus* and *C. albicans* from tissues. Thus, vaccine-mediated adaptive immunity can protect against both infections by targeting microbes for destruction by innate effectors.

## Author Summary

The bacterium *Staphylococcus aureus* and the fungus *Candida* are the second and third leading cause of bloodstream infections in hospitalized patients. A vaccine to prevent such infections would be of enormous public health benefit. The leading hypothesis to explain why vaccines have not been successfully developed against these infections is that the microbes causing the infections are highly complex, and use multiple weapons (so-called “virulence factors”) to cause disease in humans. Therefore, a vaccine targeting either infection would have to neutralize many of these virulence factors at the same time. We have been developing a vaccine that simultaneously targets both types of infections. Our vaccine is based on a single virulence factor used by *Candida*, which has a similar shape to virulence factors used by *S. aureus*. In the current study, we report that our vaccine induces specialized cells in the immune system to more effectively call in reinforcements to kill the organisms. These data demonstrate that vaccines against both organisms can be developed even if they do not work by neutralizing multiple virulence factors, and therefore open the door to a far wider array of vaccine types against both infections.

## Introduction

*Staphylococcus aureus* and *Candida spp*. are the second and third leading causes of bloodstream infections in hospitalized patients [1]. These organisms jointly cause at least 150,000 clinical bloodstream infections resulting billions of dollars of health-care expenditures and ∼40,000 deaths per year in the US alone [1]–[4]. Identification of immune mechanisms of protective adaptive immunity against these organisms is critical to lay the groundwork for development of active vaccine strategies against both organisms.

We previously reported that vaccination with the recombinant N terminus of the candidal Als3p adhesin (rAls3p-N) with aluminum hydroxide (Al(OH)_3_) adjuvant improved the survival of mice subsequently infected intravenously with lethal inocula of *Candida albicans* or methicillin resistant *Staphylococcus aureus* (MRSA) [5]–[7]. The vaccine retained efficacy against both infections in B cell deficient animals but not T cell deficient animals [6],[7]. Furthermore, adoptive transfer of CD4+ T cells but not B220+ B cells or immune serum improved the survival of recipient mice infected with both organisms [6],[7].

Although T cells are necessary for rAls3p-N vaccine efficacy, lymphocytes are not capable of directly killing *C. albicans* or *S. aureus* in culture [8],[9]. Therefore, the downstream effectors of vaccination against both organisms have remained unclear. In contrast to lymphocytes, phagocytes kill *C. albicans* and *S. aureus in vitro* [8],[10],[11] and *in vivo* [12]–[16], especially when primed with pro-inflammatory cytokines such as interferon (IFN)-γ, which is produced by CD4+ lymphocytes. Therefore, we hypothesized that the end effectors of rAls3p-N vaccine-mediated protection against bloodstream infection caused by *S. aureus* and *C. albicans* were phagocytes primed by pro-inflammatory cytokines produced by vaccine-responsive lymphocytes. We sought to elucidate fundamental requirements of protective host immunity to bloodstream infection caused by *S. aureus* and *C. albicans*.

## Results

### CD4+ lymphocyte-derived IFN-γ was necessary for vaccine efficacy in mice infected with either organism

We previously established that the rAls3p-N vaccine was not effective against *C. albicans* iv infection in IFN-γ-deficient mice [6]. We sought to determine if IFN-γ was similarly required for vaccine-mediated protection against *S. aureus*, and also to determine if CD4+ T cells were the required source of IFN-γ production to mediate vaccine efficacy against both organisms. IFN-γ-deficient mice or their wild-type, congenic controls were vaccinated with rAls3p-N plus Al(OH)_3_ (vaccinated) or Al(OH)_3_ alone (control), and boosted at three weeks. Two weeks following the boost, CD4+ splenic and lymph node lymphocytes from vaccinated or control donor mice were purified and cross-adoptively transferred to recipient mice (IFN-γ deficient donor cells were transferred to wild type recipient mice, and visa versa). As a negative control, vaccinated or control IFN-γ knockout mice were infected without undergoing adoptive transfer. Mice were infected via the tail-vein with *C. albicans* or MRSA the day following adoptive transfer.

**Figure 1:**
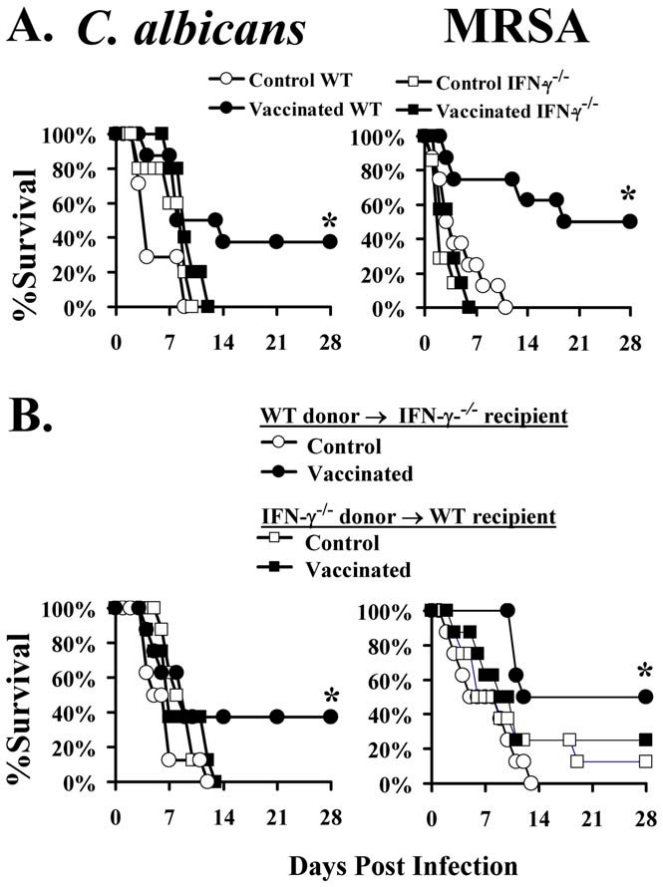
CD4 derived IFN-γ is required for vaccine protection. Wild type Balb/c mice were infected with 2×10^5^ *C. albicans* SC5314 or 2×10^7^ *S. aureus* LAC; IFN-γ deficient mice on a Balb/c background were infected with 10^5^ *C. albicans* or 10^7^ *S. aureus*. A) Wild type, n = 8 mice per group, or IFN-γ deficient mice, n = 7 mice per group, were vaccinated and infected iv via the tail-vein 2 weeks after the booster dose. *p<0.05 for wild type vaccinated vs. control by Log Rank test. B) CD4+ T cells, 5×10^6^, from vaccinated or control mice were adoptively transferred iv to recipient mice, n = 8 mice per group, 24 h prior to infection. *p<0.05 for wild type donor cells vaccinated vs. control by Log Rank test.

IFN-γ-deficient mice receiving immune CD4+ lymphocytes from vaccinated, wild type donor mice had improved survival after either infection, whereas wild type mice receiving immune CD4+ lymphocytes from IFN-γ-deficient, vaccinated donor mice did not have improved survival (Fig. 1). Cells from control donor mice were not effective at improving survival of recipient mice. Hence, IFN-γ produced by vaccine-primed CD4+ T cells was required for mediating adaptive immunity against both infections.

### Functional phagocytes were necessary for vaccine efficacy in mice infected with either organism

**Figure 2:**
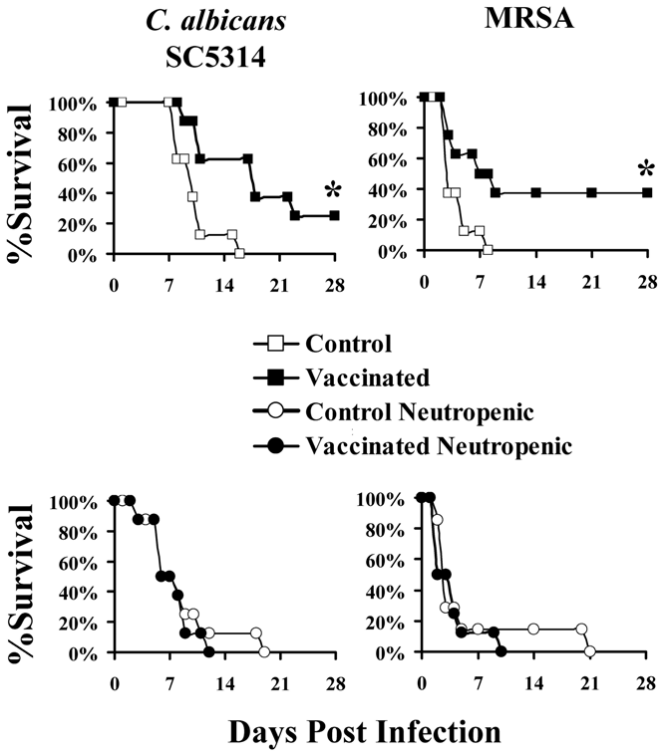
Chemotherapy-induced neutropenia ablated vaccine induced protection. Sixteen Balb/c mice per group were vaccinated with rAls3p-N plus AlOH_3_ or AlOH_3_ alone, and boosted three weeks later. Two weeks after the boost, half the mice were treated with cyclophosphamide. Two days later the mice were infected with *C. albicans* SC5314, 1.5×10^5^, *C. albicans* 15563, 7×10^5^, or MRSA LAC, 1.5×10^7^. *p<0.05 for vaccinated vs. control by Log Rank test.

Because lymphocyte derived pro-inflammatory cytokines, including IFN-γ, can activate phagocytes to mediate superior killing of *C. albicans* or *S. aureus* in culture [8], [11], [17]–[22], we sought to define the role of downstream phagocytes in adaptive immune-mediated protection. First we vaccinated mice as above and administered cyclophosphamide to induce neutropenia two weeks after the boost. Two days later we infected the mice with one of two clinical isolates of *C. albicans*, or with MRSA. The second isolate of *C. albicans* (15563) was used because it results in a less rapidly lethal infection than SC5314, and the diminished severity of infection would afford the opportunity to unmask any subtle, residual protection afforded by vaccination in the neutropenic mice. Cyclophosphamide-induced neutropenia disrupted the improvement in survival mediated by the vaccine against infections caused by either strains of *C. albicans* and *S. aureus* (Fig. 2 and Fig. S1 for the 15563 strain).

We also tested vaccine efficacy in gp91*^phox−/−^* deficient mice, the phagocytes of which are unable to generate superoxide and have marked defects in microbial killing. Such mice have been previously shown to have enhanced susceptibility to pulmonary and intraperitoneal infection by *C. albicans* [23],[24], but have not been studied in the intravenous model. We performed pilot studies to determine how susceptible to iv infection with *C. albicans* or *S. aureus* the gp91*^phox−/−^* mice were. Remarkably, we found that the 100% lethal dose (LD_100_) of *C. albicans* SC5314 was >150-fold lower in gp91*^phox−/−^* mice vs. wild-type controls (<10^3^ vs. 1.5×10^5^). The LD_100_ of *S. aureus* LAC was 2-fold lower in gp91*^phox−/^* ^−^ mice vs. wild type controls (5×10^6^ vs. 10^7^).

**Figure 3:**
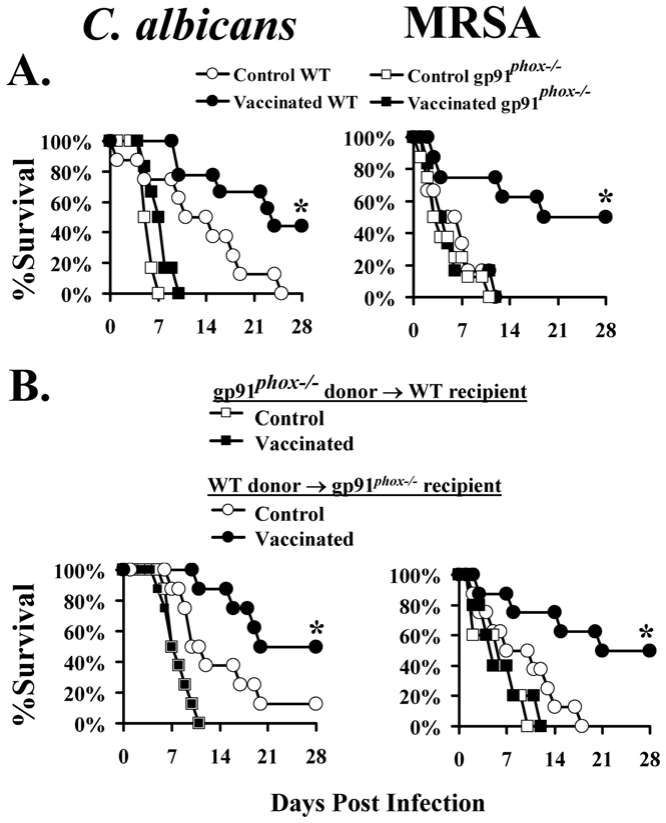
Phagocyte superoxide production is required for vaccine protection. N = 8 mice per group. Wild type C57BL/6 mice were infected with 1.5×10^5^ *C. albicans* SC5314 or 2×10^7^ *S. aureus* LAC; gp91*^phox−/−^* mice on a C57BL/6 background were infected with 1.5×10^3^ *C. albicans* or 10^7^ *S. aureus*. A) Mice were vaccinated and infected as above. *p<0.05 for wild type vaccinated vs. control by Log Rank test. B) CD4+ T cells, 5×10^6^, from vaccinated or control, wild type or gp91*^phox−/−^* mice were cross-adoptively transferred iv to recipient mice 24 h prior to infection–wild type cells transferred to gp91*^phox−/−^* mice, and visa versa. *p<0.05 for wild type donor cells vaccinated vs. control by Log Rank test.

Subsequently, gp91*^phox−/−^* and wild type mice were vaccinated with rAls3p-N plus Al(OH)_3_ or Al(OH)_3_ alone. CD4+ T cells from vaccinated or control wild type donor mice were adoptively transferred into gp91*^phox−/−^* recipient mice, and visa versa. As well, some mice were vaccinated and infected without undergoing adoptive transfer as positive (wild type) and negative (gp91*^phox−/−^*) controls for vaccine efficacy. The vaccine did not improve the survival of gp91*^phox−/−^* mice infected with either organism (Fig. 3A). While CD4+ T lymphocytes from vaccinated, gp91*^phox−/−^* donor mice improved the survival of wild type recipient mice, CD4+ T cells from vaccinated, wild-type donor mice failed to improve the survival of gp91*^phox−/−^* recipient mice (Fig. 3B).

### CD4+ lymphocyte-derived IL-17A was also necessary for vaccine efficacy

**Figure 4:**
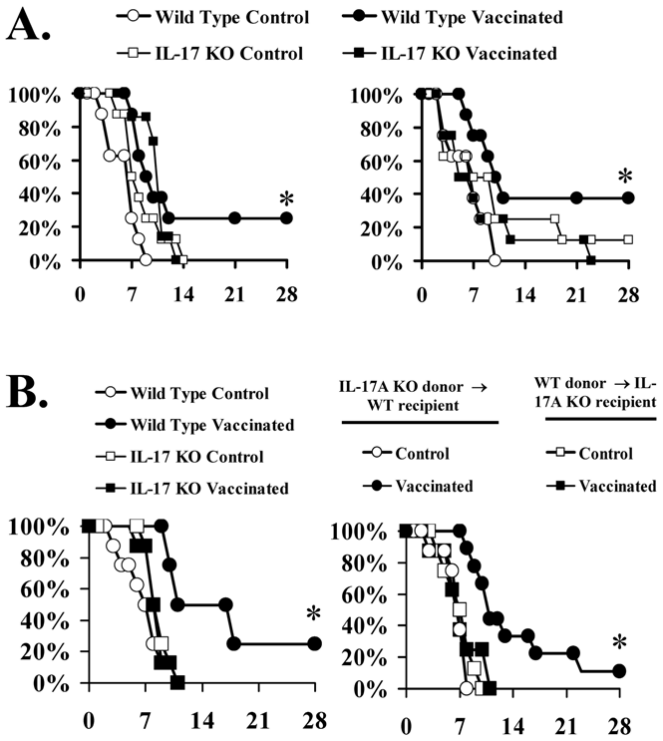
CD4+ T cell derived IL-17A was required for vaccine protection. A) Balb/c or IL-17A deficient mice on a Balb/c background (n = 8 per group) were vaccinated with rAls3p-N plus Alhydrogel or Alhydrogel alone, with a boost at 3 weeks. Two weeks after the boost, all mice were infected with 2.5×10^5^ *C. albicans* SC5314 or 2×10^7^ *S. aureus* LAC. B) Balb/c mice or IL-17A deficient mice on a Balb/c background, n = 8 per group, were vaccinated with rAls3p-N plus Alhydrogel or Alhydrogel alone. Two weeks after the boost, splenic and lymph node CD4+ T cells, 5×10^6^, from vaccinated or control, wild type or IL-17A-deficient mice were cross-adoptively transferred iv to recipient mice, wild type donor to IL-17A deficient recipient, IL-17A donor to wild type recipient, 24 h prior to infection with *C. albicans* SC5314, 2.5×10^5^ inoculum. *p<0.05 for wild type donor vaccinated vs. control by Log Rank test.

The need for downstream functional phagocytes to mediate vaccine efficacy suggested that Th17 cells, which are known to act by recruiting phagocytes to the sites of infection [25],[26], might play a role. To determine the requirement for IL-17 and Th17 cells in mediating vaccine efficacy, we vaccinated mice deficient in IL-17A, or their wild type congenic control mice. IL-17A-deficiency abrogated vaccine-mediated efficacy (Fig. 4A). Of note, in contrast to IFN-γ deficiency, IL-17A deficiency did not exacerbate the severity of infection in unvaccinated mice (comparing survival of unvaccinated, deficient vs. wild type mice).

To determine if CD4+ T cells were the primary source of IL-17A in mediating vaccine efficacy, CD4+ T cells from vaccinated or control mice were cross-adoptively transferred into recipient mice (IL-17A-deficient donor cells transferred to wild type recipient mice; wild type donor cells transferred to IL-17A-deficient recipient mice). We also repeated the survival study in wild type and IL-17A deficient mice that did not undergo adoptive transfer to serve as positive and negative controls for the adoptive transfer study. Mice were infected the day after adoptive transfer. Once again, the vaccine improved the survival of the positive control wild type mice but not the negative control IL-17A deficient mice (Fig. 4B). Adoptive transfer of CD4+ cells from vaccinated wild type donor mice improved the survival of IL-17A-deficient recipient mice (Fig. 4B). In contrast, transfer of CD4+ T cells from vaccinated IL-17A-deficient donor mice to wild type recipient mice failed to improve survival (Fig. 4C), confirming that CD4+ T cell derived IL-17A was necessary to mediate vaccine efficacy.

### Vaccination induced Th1, Th17, and Th1/17 cells in mice

**Figure 5:**
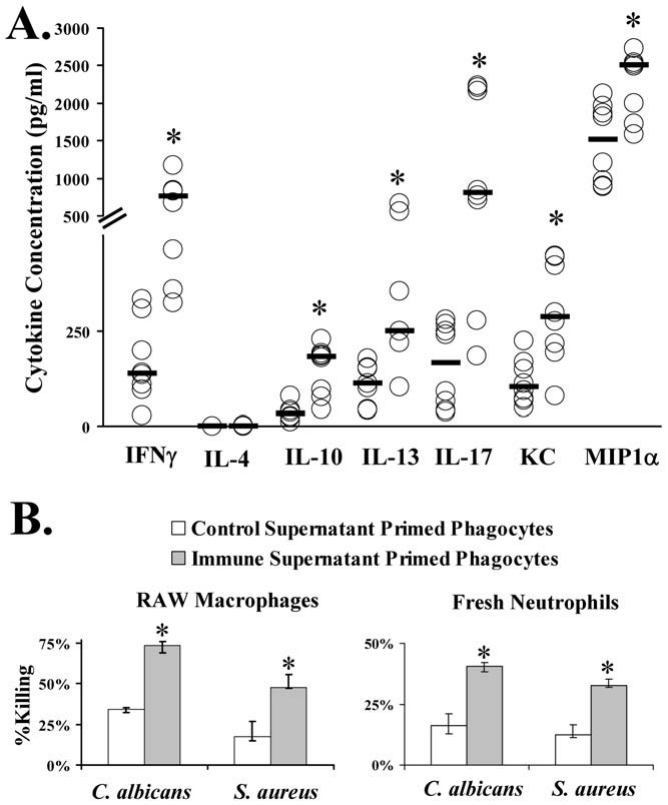
Vaccination primed lymphocytes to produce pro-inflammatory, Th1/Th17 cytokines which enhanced phagocytic killing of both organisms. A) Balb/c mice, n = 8 per group, were vaccinated with rAls3p-N plus Alhydrogel or Alhydrogel alone = Control. Two weeks after the boost splenocytes and cervical and axillary lymph node cells were harvested and incubated for 5 days with rAls3p-N. Supernatant was harvested for determination of cytokine levels. Median and interquartile ranges are shown. *p<0.03 vs. Control. B) RAW murine macrophage cells or freshly harvested murine neutrophils were primed with the above supernatants for four hours prior to incubation for one additional hour with *C. albicans* SC5314 (20∶1 RAW to *C. albicans*; 10∶1 neutrophils to *C. albicans*) or *S. aureus* LAC (5∶1 RAW to *S. aureus*; 10∶1 neutrophils to *S. aureus*). The culture wells were overlaid with agar and colonies counted the following day. Percent killing reflects reduction in colony forming units compared to number of organisms added to the wells. Median and interquartile ranges are graphed. *p<0.05 for immune vs. control supernatant.

To define the populations of cells induced by vaccination, spleens and lymph nodes were harvested from vaccinated and control mice two weeks following the boost. The cells were stimulated *ex vivo* for 5 days with rAls3p-N. Analysis of supernatants confirmed that cells from vaccinated mice produced significantly more IFN-γ and IL-17, as well as the neutrophil-acting chemokines, KC and MIP-1α, than did cells from control mice (Fig. 5A). IL-4 levels were not detectable in any supernatant from control cells; levels were detectable at low levels (< 2 pg/ml) in supernatants from 4 of the 8 mice in the vaccinated group. However, IL-10 and IL-13 levels were higher in supernatants from vaccinated than control mice. Levels of TGF-β and IL-6 were low and not significantly different in supernatants from vaccinated or control mice. Supernatants from stimulated, immune cells markedly enhanced phagocytic killing of *C. albicans* and *S. aureus ex vivo*, compared to supernatant from control cells (Fig. 5B).

**Figure 6:**
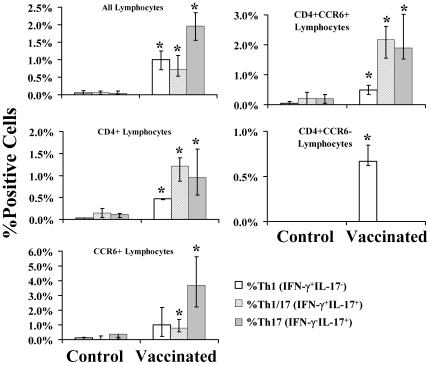
Vaccination primed Th1, Th17, and Th1/17 cells in draining lymph nodes. Balb/c mice, n = 4 per group, were vaccinated with rAls3p-N plus Alhydrogel or Alhydrogel alone = Control. Two weeks after the boost cervical and axillary lymph node cells were harvested and incubated for 5 days with rAls3p-N, followed by 6 h with PMA/ionomycin and monensin. Cells were fixed, permeabilized, and stained for CD4, CCR6, IFN-γ, or IL-17. Intracellular cytokine analysis was conducted by flow cytometry. “All Lymphocytes” were gated based on size by FSC and density by SSC; CD4+ and CCR6+ lymphocytes were further gated from the “All Lymphocyte” population by fluorescence signal indicating surface expression of these markers. Median and interquartile ranges are shown. *P<0.05 vs. Control.

Intracellular cytokine analysis of the stimulated cells demonstrated that vaccination resulted in increased frequencies of Th1 (CD4+IFN-γ+), Th17 (CD4+IL-17+), and Th1/17 (CD4+IFN-γ+IL-17+) cells in draining lymph nodes lymphocytes (Fig. 6 and Figs. S2 and S3) compared to the frequencies in unvaccinated mice. Murine CD4+CCR6- cells were enriched for the Th1 phenotype, and CD4+CCR6+ cells were enriched for the Th17 phenotype. However, a substantial proportion of CCR6+ splenocytes, and particularly CD4+CCR6+ lymphocytes, were Th1/17 (IFNγ+IL-17+) cells. The Th1/17 phenotype was predominantly found in CD4+CCR6+ cells, not in the CD4+CCR6- cells.

### Vaccination resulted in enhanced phagocyte recruitment and inflammatory cytokine production in the kidneys during *C. albicans* and *S. aureus* iv infection

To confirm the *in vivo* biological relevance of the *ex vivo* lymphocyte phenotypes, vaccinated or control mice were infected via the tail vein with *C. albicans* or *S. aureus* 2 weeks following the boost. At day 4 post-infection (the day before control mice were anticipated to begin dying), burden of infection and cytokine levels in homogenates of individually marked kidneys (primary target organ) were determined. Levels of myeloperoxidase (MPO), which is constitutively expressed at the protein level in neutrophils and has been extensively used in previous studies to quantify neutrophil influx into tissues during infection and inflammation [27]–[31], were also measured.

**Figure 7:**
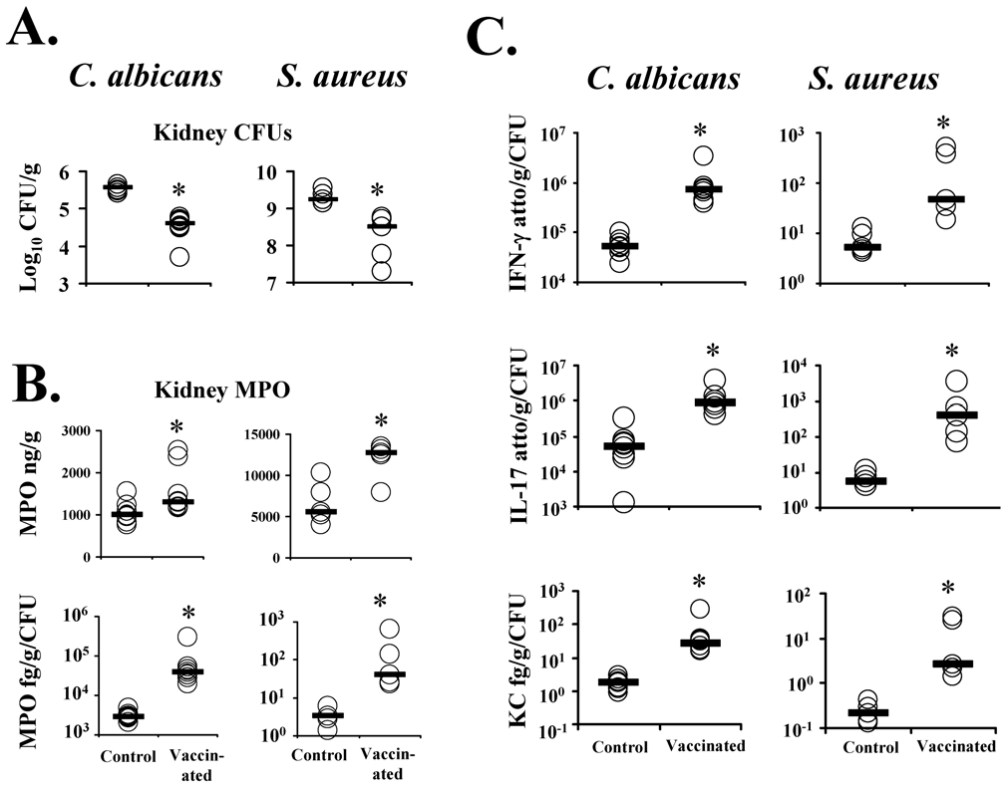
Vaccination reduced infectious burden and stimulated neutrophil influx by MPO and pro-inflammatory cytokine levels in kidneys. Balb/c mice, n = 8 per group, were vaccinated with rAls3p-N plus Alhydrogel or Alhydrogel alone. Two weeks after the boost, mice were infected iv with *C. albicans* SC5314, 2×10^5^, or *S. aureus* LAC, 3×10^7^. Four days after infection, individually marked kidneys (primary target organ of infection for both models) were harvested, homogenized, and quantitatively cultured (A). MPO levels (B) and cytokine levels (C) in organ homogenates were measured by ELISA. MPO levels are shown both as raw values in ng/g of kidney tissue, and also normalized to burden of infection in each individually marked organ in fg/g tissue/CFU per organ. Cytokine levels are shown normalized to organ CFU burden. Median and interquartile ranges are shown. *p<0.05 vs. Control by Mann Whitney U test.

Vaccination resulted in a ∼10-fold reduction in kidney fungal burden and ∼5-fold reduction in kidney bacterial burden (Fig. 7A). MPO levels were increased in vaccinated mice relative to control mice infected with either organism (Fig. 7B). A recent study reported a 95% correlation between organ fungal burden and neutrophil influx in mice infected with different strains of *C. albicans* or *C. dubliniensis* [32]. Therefore, any enhanced neutrophil influx resulting from vaccination could be offset by the diminished stimulus for neutrophil influx caused by reduced fungal burden in the vaccinated mice. To isolate the impact on MPO levels of vaccination, and not severity of infection, we adjusted absolute MPO levels in individually marked organs for the fungal or bacterial burden in those individual organs. Vaccination resulted in a marked increase in neutrophil influx relative to the infectious burden of organism in the tissues (Fig. 7B). By histopathology, the inflammatory infiltrate was predominantly neutrophilic, with scattered foci of macrophages. Semi-quantitative scoring of histopathology sections by a blinded pathologist to estimate neutrophil influx into tissues was concordant with the quantitative MPO levels.

Concordant with *ex vivo* cytokine measurements, absolute levels of IFN-γ, IL-17, and the neutrophil-acting CXC chemokine, KC, were higher in the kidneys of vaccinated versus control mice (p<0.05 for all comparisons of vaccinated vs. control levels for all three cytokines, in mice infected with *C. albicans* or *S. aureus*). After adjusting for infectious burden in individual organs, vaccination markedly increased cytokine levels relative to infectious burden (Fig. 7C).

**Figure 8:**
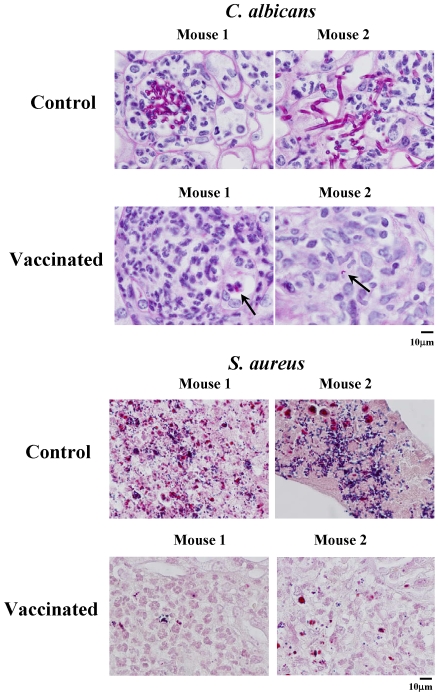
Unvaccinated mice had less neutrophil influx relative to fungal and bacterial burden than vaccinated mice. Representative histopathological sections from kidneys from 2 mice per group are shown. Control mice infected with *C. albicans* had multiple abscesses with visible hyphae and pseudohyphae throughout the kidneys. Vaccinated mice infected with *C. albicans* had abscesses, but with far less fungus visible. Numerous abscesses were seen in both vaccinated and control mice infected with *S. aureus*. However, overall the abscesses in the control mice infected with *S. aureus* had considerably more bacteria by gram stain than the abscesses in the vaccinated mice. Sections were stained by PAS (for *C. albicans*) or H&E (to show the neutrophil influx and the extent of tissue necrosis) and Gram stain (to show *S. aureus* dark purple clusters). Magnification = 1000×.

Histopathology confirmed a marked increase in organism burden in the vaccinated mice versus control mice infected with either organism (Fig. 8). Numerous microabscesses with hyphal and pseudohyphal elements were scattered throughout the kidneys of control mice infected with *C. albicans*. Microabscesses were also found in the kidneys of vaccinated mice, but most of the abscesses had no fungal elements visible, and those few abscesses with fungal elements contained blastospores or small hyphal fragments. Control mice infected with *S. aureus* had large renal abscesses with numerous gram positive cocci on Gram stain. Vaccinated mice also had renal abscesses with extensive neutrophil influx, but in most abscesses fewer staphylococcal organisms were seen on Gram stain in vaccinated than control mice (Fig. 8).

## Discussion

One hypothesis regarding the failure to date to develop an effective vaccine against *S. aureus* or *Candida* has been the need to simultaneously disrupt multiple virulence factors for such complex pathogens, whereas most vaccines to date have targeted only one virulence factor [33],[34]. However, we have previously confirmed that humoral immunity is neither necessary nor sufficient for rAls3p-N vaccine-induced protection against either organism [6],[7]. Furthermore, homozygous disruption of *ALS3* in *C. albicans* does not result in a loss of pathogenicity *in vivo* in mice, so the protection mediated by the rAls3p-N vaccine is not the result of abrogation of Als3p virulence functions. The current study confirms that vaccination can be effective by targeting the organism for destruction by increasing the quantity and microbicidal functions of innate phagocytic effectors at the site of infection, irrespective of affecting virulence functions in the organism. Therefore, potential vaccine antigens need not be restricted to microbial virulence factors, and can be expanded to include any target antigen which results in a potent Th1 and/or Th17 immune response against the organism. These data are concordant with the established role of Th17 cells in mediating protection following immunization of mice against *Mycobacterium tuberculosis*, *Helicobacter pylori* and *Pseudomonas aeruginosa* [31],[35],[36].

In unvaccinated animals, deficiency in IFN-γ but not IL-17A exacerbated the severity of iv infection caused by both *S. aureus* and *C. albicans*. These results are concordant with recent studies demonstrating that IL-17-deficient mice were not more susceptible to bloodstream infection caused by *S. aureus* [37] or invasive gastric infection caused by *C. albicans* [38]. Furthermore, a recent study reported that abrogation of the dectin-2 receptor blocked Th17 induction by *C. albicans* in mice, but despite the lack of a Th17 response did not affect the ability of mice to clear fungus from tissue during systemic infection [39]. Collectively, these results indicate that Th17 cells/IL-17A are not necessary for normal murine host defense against disseminated candidiasis.

In contrast, IL-17 has been shown to be critical for host defense against cutaneous and oropharyngeal infections caused by *S. aureus* [37] and *C. albicans* [40], respectively. Furthermore, our results are discordant with the previous finding that IL-17 receptor-deficiency moderately exacerbated the severity of bloodstream infection caused by *C. albicans* [41]. The lack of a requirement for IL-17A to mediate normal host defense against disseminated candidiasis likely reflects the ability of IL-17F, which also activates the common IL-17 receptor, to complement an IL-17A deficiency. Differences in *C. albicans* infecting strain and mouse host strain may also account for differences between our study and the prior. However, a critical point is that IL-17F could not compensate for the requirement for IL-17A in mediating protective, vaccine-induced, adaptive immunity, since IL-17A deficiency abrogated vaccine efficacy.

We confirmed that the rAls3p-N vaccine specifically primed splenic and lymph node lymphocytes to produce high levels of both IFN-γ and IL-17, as well as the neutrophil chemokines, KC and MIP-1α (the latter of which is chemotactic for both mononuclear cells and neutrophils [42]–[47]). The predominant IFN-γ expression in lymph nodes was found in CCR6- Th1 cells which did not produce IL-17 (CD4^+^CCR6^−^IFN-γ^+^IL-17^−^), and the predominant IL-17 expression in lymph nodes was found in CCR6+ cells. However, we also found substantial numbers of Th1/17 cells, which met or exceeded the frequency of Th17 cells, in the CD4+CCR6+ fraction. The Th1/17 cells were found virtually exclusively in the CCR6+ fraction, and none were found in the CD4+CCR6- fraction. Recent studies have indicated that yeast mannosylated proteins prime Th17 cells via activation of the mannose receptor [48], and that O-linked mannoproteins can activate IFN-γ production via ligation of TLR4 [49]. Since the rAls3p-N protein has O-linked yeast high mannose groups, co-ligation of the mannose receptor and TLR4 on antigen presenting cells may enable induction of Th1, Th17, and Th1/17 cells. The role of specific antigen presenting cells in priming lymphocytes for Th1, Th17, or dual Th1/17 responses is currently under investigation.

We found variations in the total number of surviving mice from experiment to experiment, ranging from as high as 87% to as low as 12.5%. Variations in outcome are most likely accounted for by variations in infectious inoculum and infecting strain. Our challenge model, using the standard SC5314 clinical isolate of *C. albicans*, is extremely rigorous, and is considerably more rigorous than challenge with other clinical strains of *C. albicans* [6],[50],[51], as evidenced by the superior efficacy seen in the current study with another clinical bloodstream isolate of *C. albicans* (15563). We have previously shown that mice infected with the inocula of SC5314 used in these experiments die of overwhelming septic shock [52]. Candidal septic shock causes >50% mortality in humans despite treatment with antifungal therapy [4]. Hence, achievement of survival approaching 50% by vaccination alone is felt to reflect meaningful protection. Furthermore, the experiment in which 12.5% survival was seen in the vaccinated arm was an adoptive transfer experiment, in which immune cells from wild type mice were transferred into IL-17A-/- recipient mice. Thus, while IL-17A production from CD4+ immune T cells can transfer protection, production of IL-17A by other cell types may be required to achieve maximal protection. Specifically, we previously found that immune CD8+ T cells could transfer protection against *S. aureus* [7], and macrophages or dendritic cells can produce pro-inflammatory cytokines such as IFN-γ, suggesting that these cell types may play an adjunctive role and be required for full vaccine-mediated protection.

We previously reported that cyclophosphamide-induced neutropenia did not completely abrogate vaccine-induced protection during subsequent disseminated candidiasis [53]. In contrast, in the current study, we did find total abrogation of protection against both candidal strains and against *S. aureus*. The prior study used a different but related vaccine immunogen, rAls1p-N, instead of rAls3p-N. As well, the prior study used Complete Freund's Adjuvant (CFA), not Al(OH)_3_. The greater efficacy of the former adjuvant may account for the residual efficacy found in neutropenic mice in the former study.

*S. aureus* and *C. albicans* express adhesins on their cell surface which possess similar three dimensional shapes [54] and which bind to similar endovascular surfaces (e.g. endothelial cells and subendothelial matrix proteins) and medically relevant plastics [54],[55]. Given these similar virulence mechanisms, it is not surprising that the organisms also infect patients with similar risk factors, including post-operative and trauma patients, patients with central venous catheters, patients on hemodialysis, and patients with compromised phagocytic host defense mechanisms [4],[56],[57]. Finally, our data demonstrate that the host defends itself against both infections by similar mechanisms, and that adaptive immunity to both organisms required CD4+ T cell production of both IFN-γ and IL-17A.

In summary, the rAls3p-N vaccine improved outcomes in mouse models of iv *S. aureus* and *C. albicans* infection by inducing upstream, pro-inflammatory, Th1, Th17, and Th1/17 lymphocytes, which enhanced recruitment and activation of neutrophils in infected tissues, thereby reducing tissue infectious burden. Thus, vaccination showed a potential to protect against both infections by targeting the microbes for enhanced destruction by innate effector cells, irrespective of neutralization of microbial virulence factors. Therefore, potential vaccine antigens need not be restricted to microbial virulence factors, and can be expanded to include any target antigen which results in a potent Th1 and/or Th17 immune response against the organisms.

## Methods

### Organisms and mouse strains

*C. albicans* SC5314 was supplied by W. Fonzi (Georgetown University), and *S. aureus* LAC, a USA300 MRSA clinical isolate, was provided by Frank Deleo (NIAID/NIH). *C. albicans* 15563 is a clinical bloodstream isolate from a patient at Harbor-UCLA Medical Center which is also virulent in our murine model [50]. *Candida* was serially passaged three times in yeast peptone dextrose broth (Difco) at room temperature prior to infection. *S. aureus* was grown overnight at 37°C in BHI broth, and then passaged for 4 hours at 37°C in fresh BHI broth.

Female Balb/c or C57BL/6 mice were obtained from Taconic Farms (Bethesda, MD). Congenic IL-17A deficient mice on a Balb/c background were obtained from Y. Iwakura (University of Tokyo) [58]. Vaccinated mice were infected via the tail vein with the appropriate inocula of *C. albicans* blastospores or *S. aureus* organisms in PBS, as previously described [7],[52]. In some experiments, mice were made neutropenic by treatment with 230 mg/kg cyclophosphamide 2 days prior to infection, a regimen which results in profound neutropenia for approximately one week [59],[60]. All procedures involving mice were approved by the Los Angeles Biomedical Research Institute animal use and care committee, following the National Institutes of Health guidelines for animal housing and care.

### Immunization protocols

rAls3p-N (amino acids 17 to 432 of Als3p) was produced in *Saccharomyces cerevisiae* and purified by Ni-NTA matrix affinity purification as previously described [61]. Mice were immunized by subcutaneous (SQ) injection of 300 µg of rAls3p-N in 0.1% Al(OH)_3_ (Alhydrogel, Brenntag Biosector, Frederikssund, Denmark) in PBS. Control mice received adjuvant alone on the same schedule. Some mice were boosted at 21 days. Mice were infected two weeks following the boost.

### Adoptive transfer and passive immunization

Serum and splenic lymphocytes were harvested from vaccinated or control mice, as we have previously described [62]. Lymph node lymphocytes were harvested from cervical and axillary lymph nodes, based on pilot studies with Evans Blue dye lymph node mapping demonstrating that SQ vaccination at the base of the neck drained primarily to these lymph nodes. For adoptive transfers, splenic and lymph node lymphocytes were pooled. CD4+ T lymphocytes were purified by use of the IMag system (BD Pharmingen), as we have described [6],[7]. Purified lymphocytes (5×10^6^ per mouse) were administered iv to congenic, unvaccinated recipient mice. Transferred cell populations were ≥95% pure by flow cytometric analysis. Mice were infected via the tail-vein with *C. albicans* SC5314 24 h after lymphocyte adoptive transfer.

### Intracellular cytokine analysis and cytokine supernatant analysis

Intracellular cytokines from lymphocytes were analyzed based on a modification of our previously described method [62]. In brief, cervical and axillary lymph nodes and spleens were dissected from vaccinated or control mice and passed through 70 µm filters. Cells were stimulated for 5 days with rAls3p-N (12.5 µg/ml) in complete media (RPMI 1640, 50 U/ml penicillin, 50 µg/ml streptomycin, 2 mM L-glutamine, 10% FBS, 5 µM 2-ME) in 96 well plates. PMA (50 ng/ml), ionomycin (1 µM), and monensin (10 µg/ml) were added during the final 6 hours of culture. Supernatant was harvested prior to adding monensin for analysis of cytokine content using Cytometric Bead Array Flex kits (BD Pharmingen, La Jolla, CA) or ELISA (for IL-6, TGF-β, and IL-13), per the manufacturer's instructions. Cells were stained on ice with PerCP-anti-CD4 and Alexa_647_-anti-CCR6 (BD Pharmingen, San Diego), or their isotype control antibodies. The cells were fixed and permeabilized as previously described [62]. Intracellular cytokines were stained with rat FITC-anti-mouse IFN-γ and PE-anti-IL-17, or their isotype controls (BD Pharmingen). Four-color flow cytometry was performed on a Becton-Dickinson FACScan instrument calibrated with CaliBRITE beads (Becton Dickinson, San Jose, CA) using FACSComp software as per the manufacturer's recommendations. Data for each sample were acquired until 10,000 CD4^+^ lymphocytes were analyzed. Th1 cells were defined as CD4^+^IFNγ^+^IL-17^−^, Th17 cells defined as CD4^+^IFN-γ^−^IL-17^+^, and Th1/17 cells defined CD4^+^IFNγ^+^IL-17^+^.

### Killing assay

The killing assay for both *C. albicans* and *S. aureus* was modified based on our well-described assay [59],[60]. In brief, RAW murine macrophage cells or murine neutrophil cells were grown in DMEM plus 10% fetal bovine serum. Fresh murine neutrophils were harvested by dextran sedimentation of whole, heparanized blood, followed by centrifugation over Ficoll Hypaque at 500 g for 10 minutes. The RAW cells or neutrophils were added into 24 well plates, the media in the wells was aspirated and the RAW cells or fresh neutrophils were cultured for 4 hours in 10% conditioned media (from vaccinated or control splenic and lymph node lymphocytes exposed to rAls3p-N for 5 days) plus 90% complete media (RPMI + 10% FBS). The conditioned media was then aspirated, and the microorganisms added to the wells in fresh DMEM plus 10% fetal bovine serum. Microorganisms were added to the wells at a ratio of 20∶1 RAW cells to *C. albicans*, 5∶1 RAW cells to *S. aureus*, or 10∶1 fresh neutrophils to *C. albicans* or *S. aureus*. Media for the wells containing *S. aureus* contained no antibiotics. The cells were incubated at 37°C for 1 h, at which point 4% blood heart infusion (BHI) agar was directly added to the wells. Plates were incubated overnight at 37°C and colony forming units (CFUs) counted in each well. Killing was defined as the percent reduction CFUs in wells containing co-cultures of phagocytes cells and microorganisms compared to wells just containing microorganisms.

### Tissue burden, whole organ cytokines, myeloperoxidase (MPO), and histopathology

On day 4 post-infection, kidneys (primary target organ) were harvested and homogenized in saline with protease inhibitors (pepstatin, leupeptin, and PMFS). For determination of infectious burden, organ homogenates were quantitatively cultured on Sabourad dextrose agar for *C. albicans* or tryptic soy agar for *S. aureus*. Whole organ cytokines were analyzed from kidney homogenates by ELISA (R&D Systems) or Cytometric Bead Array Flex kit for KC (BD Pharmingen, La Jolla, CA), per the manufacturer's instructions. MPO levels were determined by ELISA (Hycult Biotechnology, Uden, Netherlands) of whole organ homogenates. For histopathology, organs were fixed in zinc-buffered formalin, embedded in paraffin, sectioned, and stained with PAS for fungi and H&E and Gram stain for bacteria.

### Statistics

The non-parametric Log Rank test was utilized to determine differences in survival times. The Wilcoxon Rank test was used to compare cytokines, MPO levels, and organ burden across groups. P<0.05 was considered significant.

## Supporting Information

Figure S1 Chemotherapy-induced neutropenia ablated vaccine induced protection against a second *C. albicans* clinical isolate. Sixteen Balb/c mice per group were vaccinated with rAls3p-N plus Al(OH)_3_ or Al(OH)_3_ alone, and boosted three weeks later. Two weeks after the boost, half the mice were treated with cyclophosphamide. Two days later the mice were infected with *C. albicans* 15563 (7x10^5^). *p<0.05 for vaccinated vs. control by Log Rank test.
(1.28 MB TIF)

Figure S2 FACS plots for gating on Th1, Th17, and Th1/17 cells in draining lymph nodes. Shown here are representative FACS plots, corresponding to the data in Fig. 6 of the manuscript, demonstrating acquisition gates based on size (FSC), density (SSC), or expression of CD4 or CCR6 on the cell surface.
(1.85 MB TIF)

Figure S3 Vaccination primed Th1, Th17, and Th1/17 cells in draining lymph nodes. FACS plots demonstrating analysis of cytokine expression among lymphocytes using the gates shown in Fig. S2.
(1.52 MB TIF)
