# Supplementary figures and images for: Th1-Th17 Cells Mediate Protective Adaptive Immunity against Staphylococcus aureus and Candida albicans Infection in Mice
Source: PLoS Pathog. 2009 Dec 24;5(12):e1000703. doi: 10.1371/journal.ppat.1000703 (PMC2792038; doi:10.1371/journal.ppat.1000703)

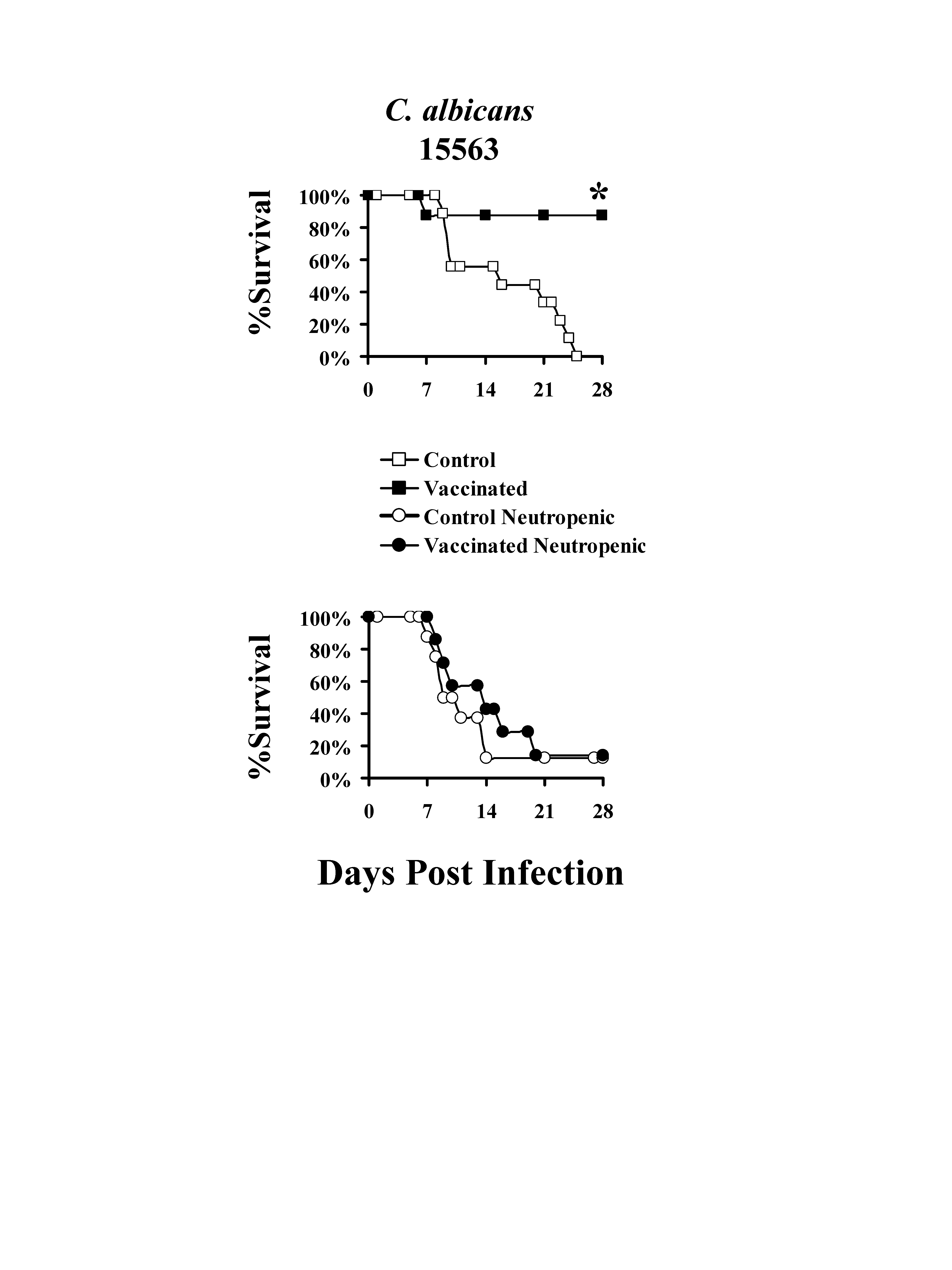

Supplement: Figure S1 — Chemotherapy-induced neutropenia ablated vaccine induced protection against a second C. albicans clinical isolate. Sixteen Balb/c mice per group were vaccinated with rAls3p-N plus Al(OH)3 or Al(OH)3 alone, and boosted three weeks later. Two weeks after the boost, half the mice were treated with cyclophosphamide. Two days later the mice were infected with C. albicans 15563 (7x10^5^). *p<0.05 for vaccinated vs. control by Log Rank test. (1.28 MB TIF) [file ppat.1000703.s001.tif]

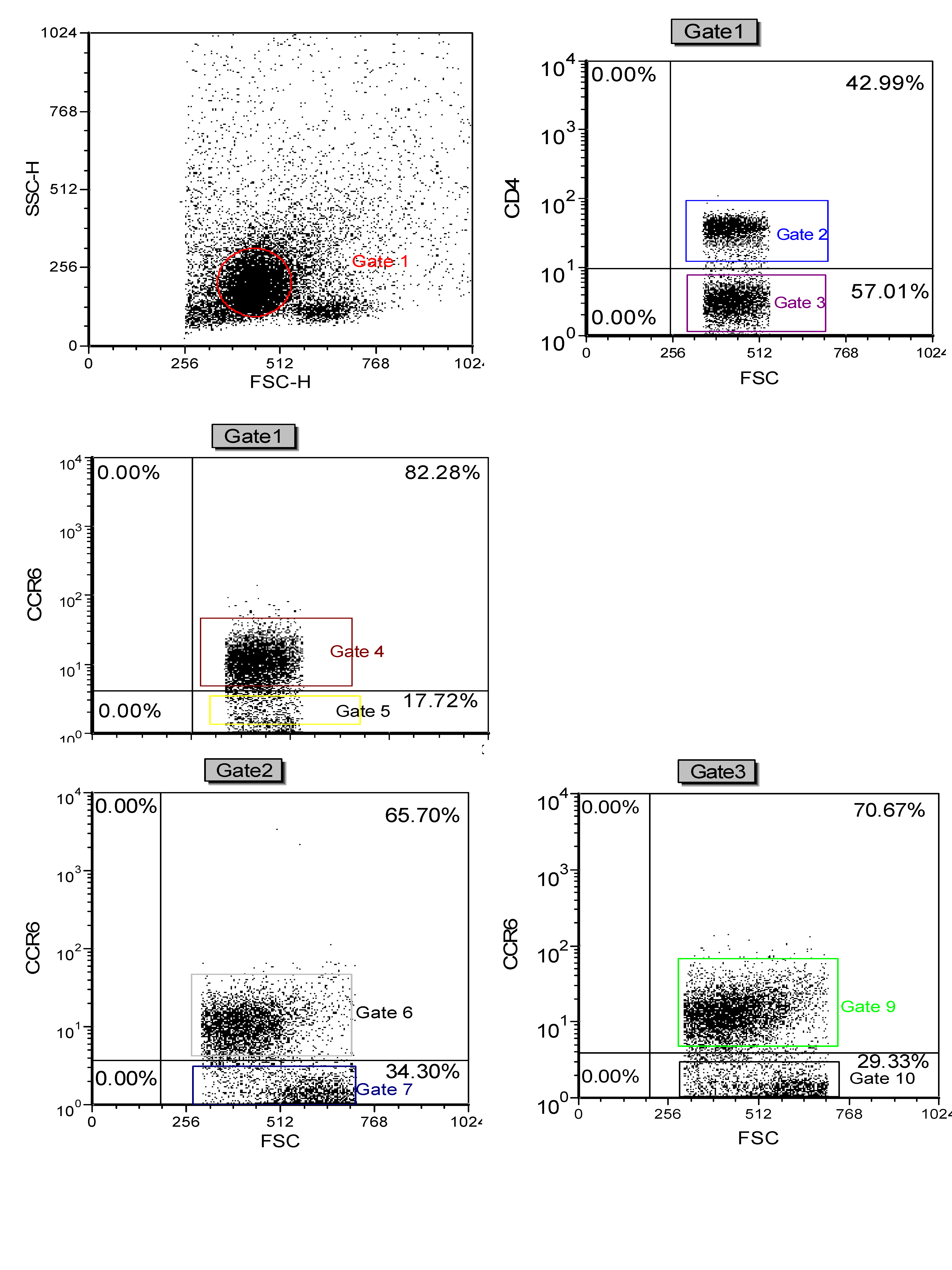

Supplement: Figure S2 — FACS plots for gating on Th1, Th17, and Th1/17 cells in draining lymph nodes. Shown here are representative FACS plots, corresponding to the data in Fig. 6 of the manuscript, demonstrating acquisition gates based on size (FSC), density (SSC), or expression of CD4 or CCR6 on the cell surface. (1.85 MB TIF) [file ppat.1000703.s002.tif]

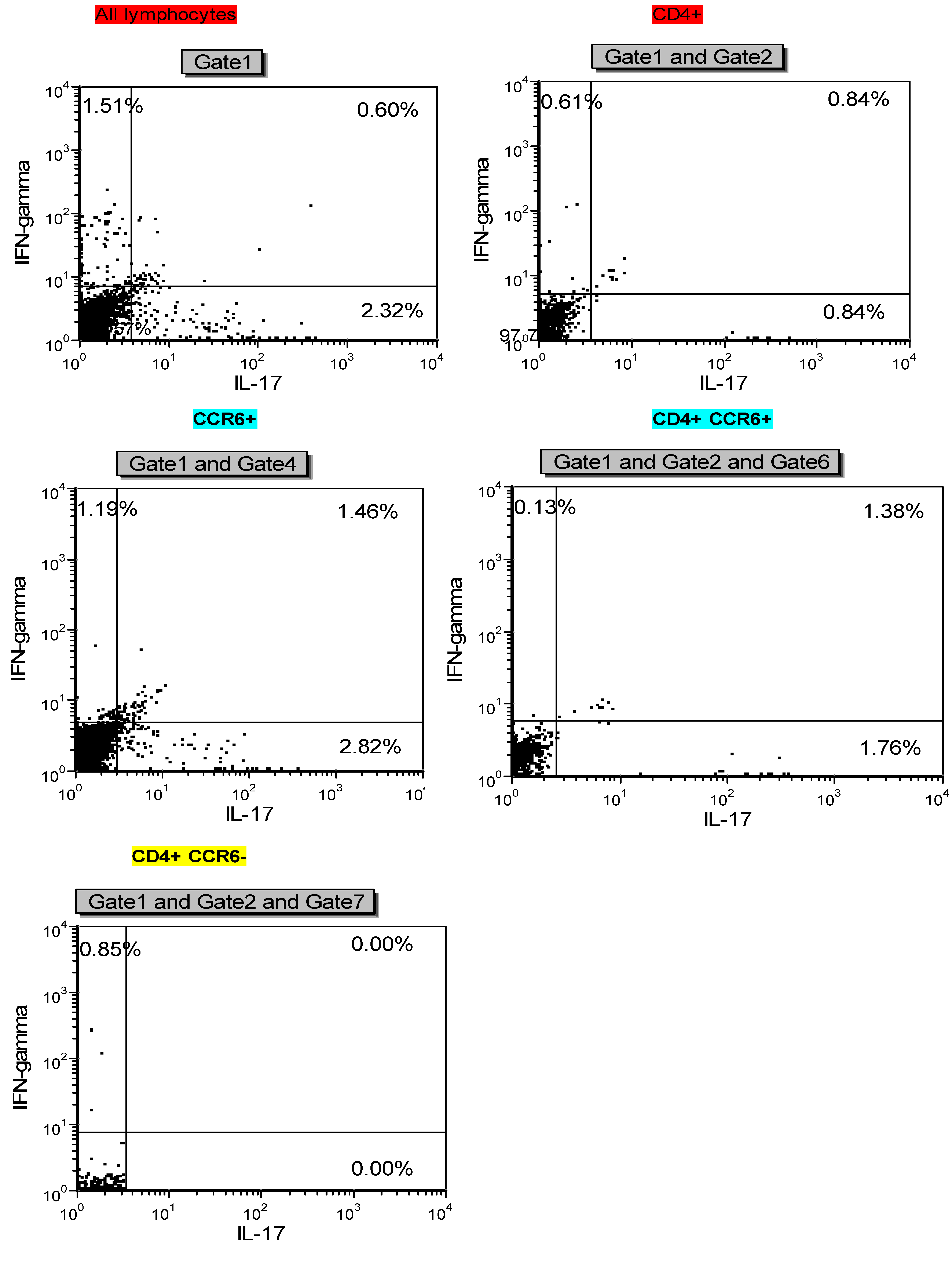

Supplement: Figure S3 — Vaccination primed Th1, Th17, and Th1/17 cells in draining lymph nodes. FACS plots demonstrating analysis of cytokine expression among lymphocytes using the gates shown in Fig. S2. (1.52 MB TIF) [file ppat.1000703.s003.tif]
